# Early reproductive investment, senescence and lifetime reproductive success in female Asian elephants

**DOI:** 10.1111/jeb.12350

**Published:** 2014-03-03

**Authors:** A D Hayward, K U Mar, M Lahdenperä, V Lummaa

**Affiliations:** * Department of Animal and Plant Sciences, University of SheffieldSheffield, UK; † Section of Ecology, Department of Biology, University of TurkuTurku, Finland

**Keywords:** ageing, antagonistic pleiotropy, disposable soma, reproductive costs, senescence, trade-off

## Abstract

The evolutionary theory of senescence posits that as the probability of extrinsic mortality increases with age, selection should favour early-life over late-life reproduction. Studies on natural vertebrate populations show early reproduction may impair later-life performance, but the consequences for lifetime fitness have rarely been determined, and little is known of whether similar patterns apply to mammals which typically live for several decades. We used a longitudinal dataset on Asian elephants (*Elephas maximus*) to investigate associations between early-life reproduction and female age-specific survival, fecundity and offspring survival to independence, as well as lifetime breeding success (lifetime number of calves produced). Females showed low fecundity following sexual maturity, followed by a rapid increase to a peak at age 19 and a subsequent decline. High early life reproductive output (before the peak of performance) was positively associated with subsequent age-specific fecundity and offspring survival, but significantly impaired a female's own later-life survival. Despite the negative effects of early reproduction on late-life survival, early reproduction is under positive selection through a positive association with lifetime breeding success. Our results suggest a trade-off between early reproduction and later survival which is maintained by strong selection for high early fecundity, and thus support the prediction from life history theory that high investment in reproductive success in early life is favoured by selection through lifetime fitness despite costs to later-life survival. That maternal survival in elephants depends on previous reproductive investment also has implications for the success of (semi-)captive breeding programmes of this endangered species.

## Introduction

Senescence may be defined as a decline in individual physiological and cellular function with increasing age. Evolutionary theories of senescence suggest that this decline occurs because the strength of natural selection weakens with age, meaning that late-acting deleterious mutations accumulate and genes that have positive effects on fitness early in life will be selected despite negative effects later in life (Medawar, 1952; Williams, 1957; Kirkwood, 1977). Studies on wild birds and mammals have shown that reproductive performance generally increases from reproductive maturity due to growth or experience, plateaus in ‘prime’ age, and then declines in later life (Nussey *et al*., 2008a). Due to limited resources for growth, reproduction and cellular repair, the rate of senescence at older ages is predicted to increase in individuals with high early-life investment in reproduction (Kirkwood & Rose, 1991). Laboratory experiments on model species and a growing number of studies on wild vertebrate populations have shown that increases in early reproductive effort, such as lower age at first reproduction or higher early-life fecundity, can result in reductions in survival and/or breeding performance in old age and more rapid senescence (Gustafsson & Part, 1990; Reid *et al*., 2003; Descamps *et al*., 2006; Nussey *et al*., 2006; Reed *et al*., 2008). For example, female collared flycatchers (*Ficedula albicollis*) subjected to experimentally enlarged broods early in life laid smaller clutches at older ages (Gustafsson & Part, 1990), and female red deer (*Cervus elaphus*) with high early-life fecundity showed stronger subsequent declines in offspring birthweight and delays in calving dates (Nussey *et al*., 2006). Such relationships among early life reproductive investment, later life survival or maternal performance and rates of senescence have also been shown to have a genetic basis in natural populations (Pettay *et al*., 2005; Charmantier *et al*., 2006; Nussey *et al*., 2008b; Wilson *et al*., 2008).

Most of the previous studies investigating effects of early-life environmental conditions or reproduction on ageing in natural populations have considered the effects of early reproductive effort on single life-history traits, but little is known of the consequences of age-related fitness variation in single traits for lifetime fitness (Bouwhuis *et al*., 2010). Typically, age-specific survival or one aspect of reproductive performance is monitored, but to capture individual variation in fitness, both survival and reproductive success, and their putative trade-off should be measured (Brommer *et al*., 2007; Jones *et al*., 2008). This shortage is despite the fact that in many natural populations, individuals may reproduce across many years under diverse environmental conditions. Without assessing the impact of early-life reproductive effort on survival and reproduction in later life, the long-term fitness consequences of early reproductive effort cannot be determined (Nussey *et al*., 2008a). Few longitudinal studies to date have attempted to investigate how the different reproductive trajectories (low vs. high early reproductive output) are selected via individual lifetime fitness (Descamps *et al*., 2006).

It is also currently unclear how the general theories of senescence apply to species with long lifespan. This is of particular importance given the prediction that in long-lived mammals, senescence should commence at an earlier age relative to the mean lifespan, be experienced by more individuals and be under stronger negative selection than in short-lived species (Turbill & Ruf, 2010; Bouwhuis *et al*., 2012). The longest lived endotherms studied to date using longitudinal lifelong datasets are birds such as mute swans *Cygnus olor* (Charmantier *et al*., 2006), common terns *Sterna hirundo* (Rebke *et al*., 2010), common guillemots *Uria aalge* (Reed *et al*., 2008) and grey-headed albatrosses *Thalassarche chrysostoma* (Catry *et al*., 2006), with maximum ages of reproduction approximating 20, 20, 30 and > 40 years, respectively. However, birds live surprisingly long compared to mammals for their body size and even the longest lived bird species appear to maintain high fitness until very close to death (Coulson & Fairweather, 2001; Rattiste, 2004). Although senescence in both survival and breeding success in birds has been detected (Jones *et al*., 2008), it is less costly than in mammals (Bouwhuis *et al*., 2012) and consequently birds are often expected to senesce at slower rates (Ricklefs & Scheuerlein, 2001), due to their slower life histories for a given metabolic rate or body size (Jones *et al*., 2008; Péron *et al*., 2010). In contrast, almost all longitudinally monitored mammal species have a typical lifespan of < 15 years, representing the low-mid range of maximum longevity found in mammals. The only long-lived mammals studied in detail so far include humans and a few marine mammals (Beauplet *et al*., 2006; Bowen *et al*., 2006; Foote, 2008; Ward *et al*., 2009). However, the unusual life history of women with menopause (Williams, 1957; Lahdenperä *et al*., 2004, 2012) makes it unclear how comparable humans are to other mammals (Helle *et al*., 2005) and studies of long-lived mammals often cannot accurately age animals born before the start of the study.

Here, we use a unique multigenerational demographic dataset on a semi-captive population of Asian elephants (*Elephas maximus*) from timber camps in Myanmar (Burma) to investigate how early life reproductive investment is associated with survival, rates of reproductive senescence and lifetime fitness. In the wild, elephants can breed into their 60s and live into their 80s (Moss, 2001). The Asian elephant is classified as Endangered in the wild on the International Union for Conservation of Nature (IUCN) Red List of threatened species, and many captive populations are also facing rapid extinction unless reproductive rates increase dramatically (Wiese, 2000). Low rates of survival and fertility in captive and semi-captive populations in Myanmar increase the demand for capture of wild elephants (Mumby *et al*., 2013b), the current rate of which is predicted to lead to the extinction of wild elephants in Myanmar in < 100 years (Leimgruber *et al*., 2008). Thus, understanding the effects of early-life reproduction on subsequent survival and reproductive performance will contribute to the welfare and conservation of the species. Our data come from state-owned elephants involved in the Myanmar timber industry whose life histories are comprehensively documented in log-books and checked by officials on a weekly basis, as stipulated by state law (Mar, 2007). Although the elephants in our dataset are semi-captive and used in the timber industry subject to set workloads, they forage unsupervised in the forest for up to 14 h per night and during official rest periods, during which time they mix and breed with captive and wild elephants. Breeding rates are thus natural and unmanaged by humans.

Previous analysis of this population has investigated the association between reproduction and immediate survival across the ontogeny and its interactions with population dynamics (Robinson *et al*., 2012). Although providing estimates of the immediate costs of reproduction, this does not inform us of the long-term effects of early life reproductive investment on performance or senescence rates in later life, or how selection acts upon different life history trajectories. This study addresses both of these questions, and further provides a detailed exposition of age-specific change in survival, fertility and calf survival by assessing individuals reproductive success at each age, rather than binning data in 5-year intervals as in the previous study (Robinson *et al*., 2012). This detailed timber elephant dataset has longitudinal life histories of known individuals, allowing: (i) comparison of age-specific patterns of individual survival, reproductive rate and offspring quality in elephants with differing early reproductive rates; and (ii) determination of selection on early life reproductive investment by comparing the lifetime fitness of females with differing early reproductive rates. The results of this study will provide a rigorous longitudinal analysis of senescence patterns and selection on life history in the longest lived animal studied to date, and will contribute valuable information in the mission to improve the survival and fertility of captive elephants to conserve the species in the wild.

## Materials and methods

### Study population and data

The Asian elephant is distributed discontinuously across the Asian continent with a total wild population of 38 500–52 500, and a further 16 000 in captivity. The largest population of captive elephants (approximately 5000) is in the timber camps of Myanmar (676 553 km^2^) where elephant draught power has been utilized extensively in timber harvesting for more than a century (Toke Gale, 1971). Approximately half of the timber elephants used in Myanmar are captive-born and of known age, and half are wild-caught (mostly at around 5 years of age, but with exact ages unknown). The elephants live in forest camps, where they are used during the day as riding, transport and draft animals. At night, the elephants forage in forests in their family groups unsupervised and encounter tame and wild conspecifics. Breeding rates are natural and not managed by humans, with most calves thought to be sired by wild bulls. Calves born in captivity are cared for by their biological mothers and allo-mothers, and are weaned at around age 4–5 (Mar, 2007). Working females are rested from mid-pregnancy (11 months into gestation) until the calves reach their first birthday (Toke Gale, 1971). They are then given light scheduled work or used as baggage or transport animals for 3–4 h per day but allowed to nurse the calves on demand. Females forage during the rest of the day to maintain body reserves for milk production. Human caretakers (mahouts) do not intervene or assist in the calving/nursing processes.

We utilize this unique and extensive demographic dataset collected from the semi-captive Asian timber elephant population of Myanmar, covering the life histories of successive generations (Mar *et al*., 2012; Robinson *et al*., 2012; Mumby *et al*., 2013a, b). These data have been compiled using the elephant log books and annual extraction reports archived and maintained by the Extraction Department, Myanmar Timber Enterprise. State ownership of thousands of elephants over half a century makes it possible to compile and transfer data of all registered elephants from the log books to a database. Data recorded for each individual include registration number and name; origin (wild-caught or captive-born); date and place of birth if captive-born; mother's registration number and name if captive-born; year, place and method of capture if wild-captured; year or age of taming; dates and identities of all calves born; date of death or last known date alive; and cause of death. The elephants have never been culled, regardless of their working or reproductive performance, and with only basic veterinary care traditionally available such as treatment of injuries, their longevity corresponds well to natural survival rates (Clubb *et al*., 2008; Mar *et al*., 2012). All state-owned elephants are subject to the same regulations set by central government for hours of work per week, working days per year, and tonnage to extract per elephant. For example, in 2010 all mature elephants (aged 17–55 years) worked 3–5 days a week (depending on weather and forage availability) 5–6 h a day (maximum 8 h) with a break at noon. All elephants finish their work season by mid-February each year, and work resumes around mid-June. The maximum tonnage of logs allowed to be dragged in a year per elephant was 400 in 2010.

The ages of captive-born elephants are exact because precise dates of birth are recorded, and this study concentrates only on the records of captive-born mothers and their offspring to have accurate data on maternal age and previous reproductive history, which are incomplete for wild-captured mothers. Limitation of the dataset to only captive-born females was also necessary because methods of capture are associated with reduced later fitness in wild-captured elephants (Mar, 2007) and we did not wish to bias our results through including wild-captured individuals into the study sample. Thus, the sample analyzed here consists of all reproductive females (*n* = 416; born 1941–1990) living within eight regions (a total of 14) in Myanmar, namely Ayeyarwady, Bago, Chin, Kachin, Magway, Mandalay, Sagaing and Shan (Mar *et al*., 2012), which reproduced between 1959 and 2002. The youngest female breeding in the sample was 5 years old and the oldest was 53 years old (mean age at first birth: 19.48; median: 19 years), with the maximum lifetime number of calves 10. In this population, 26% of all calves born failed to reach their fifth birthday (Mar *et al*., 2012). These life history patterns mirror those data found for wild populations of Asian and African elephants (*Loxodonta africana*) (Moss, 2001; Sukumar, 2003; Moss *et al*., 2011).

### Statistical analyses

#### Whole-life analysis of annual breeding success

We first examined changes in female annual breeding success between ages 5–50 to identify patterns of offspring production with age. Annual breeding success was scored as a binomial trait (0 = did not produce a calf in a given year of life; 1 = produced at least one offspring), and analyzed using generalized linear mixed-effects models (GLMMs) with binomial errors and a logit link function in the R package ‘lme4’ (Bates *et al*., 2012). The data constituted 12 789 records from 416 reproductive females collected across 56 years (1946–2002). Three hundred and twenty-three of these individuals did not reach the end of their natural lifespan during the course of the study, either because they did not die before the end of the study period, or because they were moved to a different camp, and so were censored. We used the data available on exactly known ages, known censoring, and known ages at last observation to account for differences between censored and dead individuals, and between individuals who were recorded up to varying ages. This may not represent an exact estimation of individual-level ageing as described by recently developed statistical methods (Cam *et al*., 2002; van de Pol & Verhulst, 2006; van de Pol & Wright, 2009; Rebke *et al*., 2010; Reid *et al*., 2010), but these methods are unsuitable with our data structure for reasons outlined in the Discussion. Despite the fact that the following analyses may not describe exact within-individual ageing patterns, our results remain the most comprehensive analysis of age-specific reproductive success in any long-lived nonhuman mammal outside captivity to date.

Our models included year as a random effect to account for repeated measures and variation between years. Initial models fitted a random effect of individual identity, but it was found to be negligible, and so we excluded it. We firstly constructed a ‘base’ model, which contained fixed effects of the area a female lived in as a categorical variable (eight levels); whether or not an individual was censored (0 = died before the end of the study, 1 = was censored); linear and quadratic terms for age at first reproduction to determine whether the age of onset of breeding was associated with probability of breeding; linear and quadratic terms for age at last observation. Thus, all age-specific estimates account for the fact that some individuals are censored and that some are recorded for longer than others. We simplified this model by sequentially removing the least significant variables as determined by Wald *z* statistics, to return a final base model to which we subsequently added terms describing variation in annual breeding success with age. We began by fitting polynomials of age of increasing complexity (linear, quadratic, cubic), and then fitted threshold, or piecewise regression, models. We first fitted models with a single threshold, with annual breeding success changing as a linear function of age on either side of the threshold. We compared models where the threshold varied at 1-year intervals. We then considered two-threshold models, where annual breeding success changed as a linear function of age in three stages (for instance: an increase, a plateau and a decline). Again we varied the locations of the thresholds at 1-year intervals. We compared the AIC values of these ageing models, selecting the models with the lowest AIC value as that best describing the change in annual breeding success with age. In these models, age, age at first reproduction and age at last observation were divided by 100 in order to aid model convergence.

#### Effects of early life fecundity on later-life fitness

The whole-life analysis of annual breeding success suggested that it declined from the age of 19 onwards (see Results). We used the results of the above model to investigate how early life fecundity was associated with later-life survival and reproductive success by determining how reproduction before age 19 was associated with survival, fecundity and offspring survival following age 19. We recognize that individual variation exists in the onset age of senescence (Weladji *et al*., 2008; Martin & Festa-Bianchet, 2011). However, because such individual-level variation is difficult to determine for individuals with few or no reproductive events, for the purpose of this study, individual later-life senescence rates were studied with respect to their reproduction before the population average age at peak fecundity (age 19), in line with the previous studies (Nussey *et al*., 2006; Reed *et al*., 2008; Bouwhuis *et al*., 2010).

First, we analyzed the effects of early life fecundity on survival from age 19 onwards. We used Cox proportional hazards models fitted using the R package ‘survival’. We compared models which fitted different survival functions for individuals with different early-life fecundity levels: the first had the same survival pattern for individuals with early-life fecundity = 0, 1 and 2; the second compared survival patterns of individuals with early life fecundity of 0, 1, and 2; the third compared individuals with early-life fecundity of 0 vs. those with 1 or 2 calves in early life; the fourth compared individuals with early life fecundity of 0 or 1 vs. those with 2 calves in early life. We compared models using likelihood ratio tests (LRTs), comparing the log likelihoods of the models, where the *χ*^2^ test statistic was calculated as −2*(LogLik_model1_ − LogLik_model2_), with a *P*-value calculated from this statistic on the appropriate degrees of freedom.

Second, we analyzed the annual breeding success of individuals from the age of 19 onwards. We analyzed 7044 records across 42 years from 401 reproductive females who survived at least until age 19 using GLMMs with binomial errors and a logit link function. Annual breeding success was scored as a binomial trait as in the whole-life models, and we constructed a base model including area of origin, whether or not an individual was censored, age at first reproduction and age at last observation, arriving at a final base model by simplifying it as in the whole-life models. We compared the AIC values of models with null, linear, quadratic and cubic functions of age, and then added early-life fecundity to the model as a categorical variable. We tested models where individuals were classed into two early-life fecundity groups (ELF2), where females either produced no calves before the age of 19 (ELF2 = 0) or produced one or more calves (ELF2 = 1). We also tested models where individuals were classed into three early life fecundity groups (ELF3), where females produced no calves before the age of 19 (ELF3 = 0), produced one calf (ELF3 = 1), or produced two or more calves (ELF3 = 2). This tested the hypothesis that annual breeding success after the age of 19 would be lower in individuals making a larger reproductive investment in early life. We also added interactions with age to test the hypothesis that higher early-life fecundity would be associated with accelerated reproductive senescence. We selected the model with the lowest AIC value.

Third, we analyzed the effects of early-life fecundity on late-life maternal performance, by investigating whether age and early fecundity were associated with calf survival to weaning age (5 years). Calf survival was scored as a trait of the mother (0 = calf died before age 5; 1 = survived to age 5), and we analyzed the survival of 744 calves born to 333 mothers across 42 years. We constructed a base model as for annual breeding success and then compared the same models investigating effects of age, early-life fecundity and their interaction, using AIC values.

#### Early-life fecundity and lifetime breeding success

Finally, we investigated selection on early-life fecundity by analyzing its association with ‘lifetime’ breeding success, defined as the number of calves born to a female during the period she was followed by our study (until death or last observation). We analyzed data from all 416 females, and repeated the analysis using data only from females who were followed until death (*N* = 92). We used a generalized linear model (GLM) with Poisson errors. The distribution of lifetime breeding success was overdispersed, so we corrected standard errors using a quasi-GLM where the variance is given by *φ* × *μ*, where *φ* is dispersion parameter and *μ* is the mean (Zuur *et al*., 2009). ‘Lifetime’ breeding success was fitted as the response variable, with explanatory variables of study area, linear and quadratic effects of age at first reproduction and early-life fecundity as a three-level factor. Significance of the fixed effects was assessed by dropping the terms from the model and calculating *P*-values based on *F*-statistics. We present results from the analysis of all females.

## Results

### Whole-life analysis of annual fecundity

**Figure 1:**
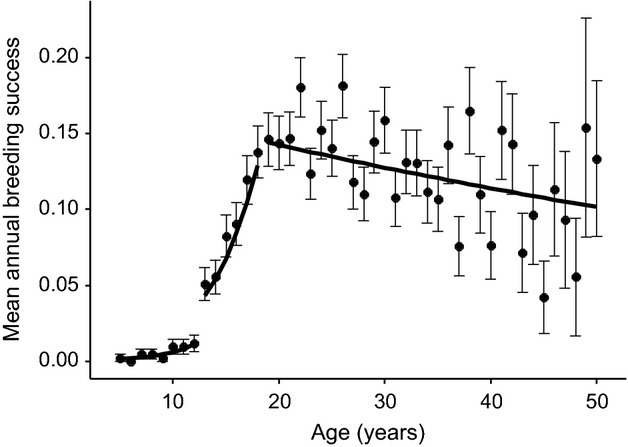
Mean annual breeding success varied significantly with age across the lifespan of female Asian elephants aged 5–50. The raw age-specific means (closed symbols ± 1 SE) suggest an initial increase, followed by a peak and subsequent decline. Also shown are predictions from Model 1218, the best fitting generalized linear mixed-effects model in Table1 (black lines). The predictions are drawn from the estimates in Table2 and are for an individual of mean age at first reproduction and last age recorded.

Our results showed clear evidence that female annual breeding success initially improved, before peaking at age 19 and subsequently declining. GLMMs including higher order polynomial age terms statistically improved model fit compared to a model without an age term (Table1: models 0–3). The model with the highest statistical support was a two-threshold model (Table1: model 1218), the parameter estimates from which are shown in Table2. Additional one- and two-threshold models are shown in the Supporting Information (Tables S1 and S2 respectively). The predictions from model 1218 showed that the ageing trajectory was comprised of three stages: low annual breeding success at ages 5–12; a rapid increase between ages 13–18; a gradual decline from age 19 onwards (Fig.1). Up to and including the age of 18, females had produced on average 0.56 ± 0.03 offspring (range = 0–3). The average number of offspring produced during the later reproductive period from the age of 19 onwards equalled 2.34 ± 0.08 (range = 0–9). Besides the effects of age, annual breeding success was higher in individuals who began reproducing earlier, as evidenced by a negative association with age at first reproduction. The base model also suggested that censored individuals had lower annual breeding success than those who were not censored (estimate = −0.18 ± 0.08, *z* = −2.31, *P* = 0.021) and that individuals who were recorded to later ages had higher annual breeding success (estimate = 2.29 ± 0.37, *z* = 6.19, *P* ≤ 0.001), suggesting that longer lived individuals were also more likely to reproduce in a given year. These were not significant in the model with age effects, but were retained to account for any variation due to their effects (Table2).

**Table 1:** A comparison of models analyzing ageing-related variation in annual breeding success in females aged 5–50. All models were generalized linear mixed-effects models (GLMMs) with binomial errors and logit link function. Models were compared using AIC values, where the best-supported model has the lowest AIC value and is shown in bold italics. ΔAIC values are shown relative to the best-supported model. Only the best one- and two-threshold models are shown; the remaining one- and two-threshold models are shown in the Supporting Information (Tables S1 and S2 respectively). Analysis was performed on 12 789 records from 416 female elephants.

(Model) structure	AIC	ΔAIC	LogLik	vs.	d.f.	*χ*^2^	*P*
(0) BASE	7628.35	552.43	−3808.2				
(1) Age	7483.12	407.21	−3735.6	0	1	147.2	<0.001
(2) Age^2^	7207.24	131.32	−3596.6	1	1	278	<0.001
(3) Age^3^	7102.93	26.02	−3542.0	2	1	107.2	<0.001
(17) Threshold = 17	7083.87	6.95	−3532.4	3	2	19.2	<0.001
***(1218) Threshold = 12 + 18***	***7076.92***	***0.00***	−***3527.0***	***17***	***2***	***10.8***	***0.005***

**Table 2:** Parameter estimates from the statistically best-supported binomial generalized linear mixed-effects models (GLMMs) for age-specific change in annual breeding success. Model 1218 from Table1 is shown, analyzing 12 789 records from 416 female elephants. Parameter estimates and standard errors (SE) are shown on the logit scale. ‘Age: AgeGroup (X)’ denotes an interaction between age as a linear covariate and age group, where the numbers in parentheses indicate the boundaries of the grouping. Note that age at first reproduction, age at last observation (LastAge) and Age were divided by 100 to aid model convergence.

Variable	Estimate	SE	*z*	*P*
Fixed effects				
Intercept	−6.3010	1.1694	−5.39	<0.001
Censored (0)	0.0000	0.0000		
Censored (1)	0.0086	0.0781	0.11	0.913
AFR	−7.5900	0.6895	−11.01	<0.001
LastAge	−0.5665	0.4588	−1.24	0.217
Age	29.4712	11.4487	2.57	0.010
AgeGroup (5–12)	0.0000	0.0000		
AgeGroup (13–18)	1.8569	1.3511	1.37	0.169
AgeGroup (19+)	6.4823	1.1651	5.56	<0.001
Age: AgeGroup (5–12)	0.0000	0.0000		
Age: AgeGroup (13–18)	−5.8405	12.2471	−0.48	0.633
Age: AgeGroup (19+)	−30.7517	11.4613	−2.68	0.007
Random effects				
ID	0.0000	0.0000		
Year	0.0125	0.0150		

### Effects of early-life fecundity on later-life fitness

The whole-life analysis of annual breeding success suggested a peak at age 19 followed by a subsequent decline. We next investigated ageing-related variation in survival and reproduction from the age of 19 onwards in relation to reproduction before age 19.

**Figure 2:**
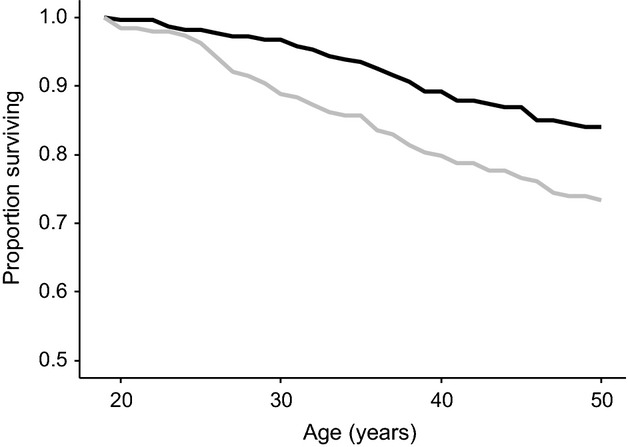
Early-life fecundity was associated with survival rates between the ages of 19 and 50. Of 213 individuals that did not reproduce before the age of 19 (black line), only 35 (16.43%) were dead by the age of 50, while 50 (26.60%) of the 188 individuals who did reproduce before age 19 (grey line) were dead by age 50. The best-fitting Cox proportional hazard model (Table2) suggested that this was a significant difference. Overall, of the 401 individuals who were alive at 19, 85 (21.20%) died before the age of 50. These survival rates to age 50 are high as all individuals included in this analysis survived to age 19, and thus had survived the period of greatest mortality (Mar *et al*., 2012).

First, early-life fecundity was negatively associated with female survival rates at older ages. Cox proportional hazards models suggested that survival after the age of 19 was higher in females that produced no calves before age 19 than in females who produced at least one calf (*β* = 0.65 ± 0.22 SE, *z* = 2.93, *P* = 0.003). This represents a hazard of 1.91 for individuals producing at least one calf before age 19 compared to those producing no calves. A comparison of these models is shown in Table S3. Of 213 females who did not reproduce by age 19, only 35 (16.43%) died between the ages of 19 and 50, whereas of the 188 females who produced at least one calf before age 19, 50 (26.60%) died before they were 50 (Fig.2).

Second, such survival costs could have been intensified by the fact that females with high early-life fecundity also attempted to produce offspring at a high rate after age 19. The statistically best-supported model for late-life annual breeding success contained a linear effect of age (Table3: model 1), suggesting a linear senescent decline in annual breeding success with increasing age (estimate = −1.51 ± 0.62). Two models suggested that higher early-life fecundity was associated with higher annual breeding success after age 19, but these effects received only marginal statistical support. A model with early-life fecundity as a two-level factor (comparing individuals who did not reproduce before age 19 with those who produced at least one calf) had a ΔAIC value of −0.24 (Table3, Model 9). This model was suggestive of a positive association between early-life fecundity and late-life annual fecundity (ELF = 1, estimate = 0.20 ± 0.13) and equated to an almost 19% higher probability of reproducing in a given year after the age of 19 in individuals who reproduced before the age of 19 vs. individuals who did not. The model including an interaction between a cubic effect of age and early-life fecundity as a two-level factor (Table3, Model 12) was also a slight improvement on Model 1, with a ΔAIC value of −0.54. These models are only suggestive of a better fit than model 1, however, as only models with ΔAIC < −2 are considered to offer a statistically better fit than a simpler model (Burnham & Anderson, 2002).

**Table 3:** A comparison of models analyzing variation in annual breeding success and calf survival in females aged 19 and over. All models were generalized linear mixed-effects models (GLMMs) with binomial errors and logit link function. Models were compared using AIC values, where the best-supported model for each trait is shown in bold italics. ΔAIC values are shown relative to the best-supported model. ELF(2) groups individuals by their early-life fecundity based on whether they reproduced in early life; ELF(3) is a factor grouping individuals based on whether they had 0, 1 or 2 or more calves before age 19 (see Materials and methods). For annual breeding success, we analyzed 7044 records from 401 reproductive females who survived at least until age 19; for calf survival, we analyzed 744 calves born to 333 mothers after the age of 19.

Model	Structure	Breeding success		Calf survival	
		AIC	ΔAIC	AIC	ΔAIC
0	Null	5526.22	3.32	842.95	22.24
1	Age	***5522.90***	***0.00***	839.51	18.80
2	Age^2^	5524.46	1.57	838.17	17.46
3	Age^3^	5526.07	3.18	830.50	9.79
4	ELF(3)	5526.74	3.84	834.68	13.97
5	ELF(2)	5526.16	3.26	844.94	24.23
6	ELF(3) + Age	5523.09	0.19	829.25	8.54
7	ELF(3) + Age^2^	5524.65	1.76	829.79	9.08
8	ELF(3) + Age^3^	5526.24	3.35	***820.71***	***0.00***
9	ELF(2) + Age	5522.66	−0.24	841.36	20.65
10	ELF(2) + Age^2^	5524.22	1.32	840.03	19.32
11	ELF(2) + Age^3^	5525.83	2.93	832.21	11.50
12	ELF(3): Age + Age	5522.35	−0.54	832.89	12.18
13	ELF(3): Age + Age^2^	5523.86	0.96	833.31	12.60
14	ELF(3): Age + Age^3^	5525.47	2.57	823.73	3.02
15	ELF(2): Age + Age	5522.97	0.07	843.28	22.57
16	ELF(2): Age + Age^2^	5524.52	1.62	841.90	21.19
17	ELF(2): Age + Age^3^	5526.16	3.26	834.18	13.47

ELF, early life fecundity.

**Figure 3:**
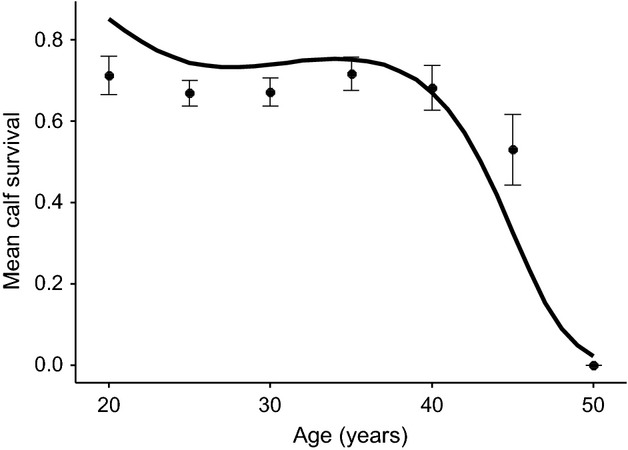
The association between female age and the survival rate of their calves to age 5. The raw age-specific means (closed symbols ± 1 SE) suggest a survival rate of calves of around 70% until the age of 40, at which point a steep decline is observed. Age groups are binned to 5-year intervals, such that 20 = ages 19–20; 25 = ages 21–25…..50 = ages 46–50. The black line shows the estimated age effect from Model 8 in Table3, with the prediction generated for an individual of mean AFR and age at last observation. The apparent discrepancy between raw data and model estimates is due to the fact that the model estimates account for variation between AFR, age at last observation and area of origin, all of which will affect the elevation of the slope.

Third, we investigated the effects of age and early-life fecundity on the ability of females aged 19 and older to raise their calf to the weaning age of 5. This is of relevance to the health of this captive population as 26% of calves die before age 5 (Mar *et al*., 2012). The best-fitting model contained a cubic effect of age and early-life fecundity as a three-level factor (Table3, Model 8). The effect of age suggested that calf survival was maintained at a high level until a maternal age of around 40 years, at which point it declined steeply (Fig.3). The effect of early-life fecundity suggested that individuals who did not produce any calves before age 19 had lower calf survival than those who produced one calf before age 19 (estimate = 1.25 ± 0.34) and those who produced two or more calves (=1.69 ± 0.60). When conditioned on mean age at first reproduction and age last seen, the model predicted a calf survival probability of 22.42% for females who did not reproduce before age 19; 50.26% for those producing one calf before age 19; 60.98% for those producing two calves.

### Early-life fecundity and lifetime breeding success

Given the opposing effects of early-life fecundity on later-life maternal survival and reproductive success, we tested for selection on early-life reproductive investment. We analyzed the association between early-life fecundity and lifetime breeding success (total number of offspring produced until death or censoring) of females with differing early-life reproductive performance. We analyzed data from all 416 females in our sample, and also restricted the analysis to the 92 females who died during the study. Results were qualitatively the same for both samples and we present the results obtained using the entire data set. A generalized linear model (GLM) revealed that females who produced one calf before the age of 19 had higher, but not statistically significantly so, lifetime breeding success than those who produced no calves before age 19 (estimate = 0.05 ± 0.06, *t* = 0.82, *P* = 0.411); those who produced two calves before age 19 had significantly higher lifetime breeding success than those who did not produce any (estimate = 0.33 ± 0.09, *t* = 3.51, *P* ≤ 0.001). On average, females who produced at least two calves before the age of 19 produced 4.77 calves in their lifetime; those who produced one calf before the age of 19 produced 3.12 calves; those who did not reproduce before the age of 19 produced 2.34 calves during their lifetime. This suggests that, despite later-life survival costs of high early-life reproduction, early reproduction is under positive selection through lifetime fitness in this population.

## Discussion

Our study on semi-captive Asian timber elephants provides rare evidence from a long-lived mammal, including humans, of age-specific fitness declines following high early-life reproductive investment. The results show that the late-life survival costs of high early-life fecundity are offset by the higher reproductive success, both in terms of offspring production and offspring survival, leading to higher lifetime breeding success and positive selection for early-life fecundity. We discuss these results in the context of empirical findings in shorter-lived species, the general predictions of life history theory, and the implications for conservation of the Asian elephant.

We found that increased early-life reproductive output was associated with reduced subsequent survival rates but greater overall lifetime fitness. This is consistent with findings in model organisms suggesting that genes associated with high early reproductive investment can be selected for even if they are detrimental later in life (Partridge & Gems, 2006). Consequently, our results might be best interpreted within the context of the disposable soma theory proposing that the compromise between allocating energy to reproduction vs. repair functions causes the body to deteriorate with age (Kirkwood, 1977). Studies on shorter-lived natural vertebrate populations have demonstrated negative associations between early reproductive investment and subsequent survival also in the wild (Gustafsson & Part, 1990; Reid *et al*., 2003; Descamps *et al*., 2006; Nussey *et al*., 2006; Reed *et al*., 2008), but the overall importance of such declines for lifetime fitness has attracted little attention (but see Bouwhuis *et al*., 2012). Previous work on this population showed that costs of reproduction were present in terms of reduced immediate survival for females who produced a recruiting calf in elephants aged > 30 years, but not in younger elephants (Robinson *et al*., 2012). However, the consequences of different reproductive strategies for longer term survival and lifetime fitness were not determined. Here, we show that in fact, high fecundity in early life does carry a survival cost, but that this cost is long-term: high early life fecundity is associated with reduced later-life survival. We also show that despite this long-tem cost, early life fecundity is favoured by selection through lifetime breeding success. That reduced survival following high early reproductive investment is also apparent in elephants, which have a long lifespan, slow reproductive rates and extended parental investment, supports the possibility that such patterns are consistent across animals with vastly differing life histories. It is also worth noting that the positive association between early-life fecundity and lifetime breeding success does not simply reflect an association between lifetime breeding success and survival. Adding lifetime breeding success to the Cox survival model of early-life fecundity shows that, as before, early-life fecundity increases the hazard of death (*β* = 1.22 ± 0.25 SE, *z* = 4.95, *P* ≤ 0.001, hazard = 3.39), but that individuals with higher lifetime breeding success have a lower hazard of death. For instance, individuals producing three calves across their lives are around half as likely to die between ages 19–50 than those with one calf (*β* = −0.69 ± 0.34 SE, *z* = −2.02, *P* = 0.044, hazard =0.50), while those producing five calves in their lifetime had an even lower hazard of death (*β* = −1.85± 0.38 SE, *z* = −4.88, *P* ≤ 0.001, hazard = 0.16). This positive association between lifetime breeding success and survival between ages 19–50 presumably exists because surviving longer enhances opportunities to breed. This research raises further questions about the life histories of such long-lived animals, such as the selection patterns on a putative menopause.

We found that survival in later life was decreased by early reproductive effort but that declines in reproductive output with age were unaffected. Indeed, early-life fecundity was positively associated with both annual breeding success and the ability to rear a calf successfully to weaning in later life. The decline in reproductive output with age began around age 20, which appears to be early for an animal that lives to > 60 years: by comparison long-lived seabirds, which may live to > 40 years, only show senescence a short time before death (Coulson & Fairweather, 2001). This result also matches the prediction that in long-lived mammals, senescence should commence at an earlier age relative to the mean lifespan than in short-lived species (Turbill & Ruf, 2010; Bouwhuis *et al*., 2012). In such species, senescence is also predicted to be experienced by more individuals and to be under stronger negative selection than in short-lived species (Turbill & Ruf, 2010; Bouwhuis *et al*., 2012). These results highlight the need to investigate the impact of early-life reproductive effort on subsequent survival and reproduction to determine the fitness implications of early-life reproductive investment (Jones *et al*., 2008). Tests for tradeoffs between early and later-life reproduction or survival often yield positive (Moyes *et al*., 2006) rather than negative (Nussey *et al*., 2006; Reed *et al*., 2008) correlations, because individuals may have both higher breeding performances across their lifespan and higher probabilities of survival (van de Pol & Verhulst, 2006), perhaps due to greater acquisition or more efficient use of resources. For example, larger females could have higher reproductive rates and longer life-expectancies, although we currently lack data on body weight for the elephants in our sample. We found that female elephants with high early reproductive investment also produced offspring at a high rate at subsequent ages but consequently suffered a survival cost. This result is important because it suggests that positive correlations between age-specific measures of reproductive performance do not necessarily imply an absence of trade-offs with other fitness components. Estimating maternal age-specific fitness by incorporating the maternal reproductive rate, offspring survival to maturity and maternal survival probability is advantageous because it encompasses several inter-related life history trade-offs, namely, current vs. future reproduction, offspring number vs. offspring quality, and maternal reproduction vs. survival. Such estimates of age-specific fitness may give a more realistic measure of the sensitivity of natural selection to genes influencing age-specific survival during the female reproductive period than previously appreciated.

Despite these results being based on a uniquely detailed longitudinal dataset from a long-lived mammal, there are caveats to the patterns described which must be acknowledged. First, changes in the population-level mean performance with age is due to two processes: within-individual change and selective mortality. The latter can create population-level change with age if individuals who are more likely to die have a level of performance which is either above or below that of individuals who survive to be measured in consecutive years (van de Pol & Verhulst, 2006). A number of statistical methods have been developed to account for selective disappearance, to determine the pattern of ageing at the individual level, but these methods are intractable for our dataset. Some of these methods use observations on lifespan to account for covariance between lifespan and breeding success (van de Pol & Verhulst, 2006; van de Pol & Wright, 2009; Reid *et al*., 2010) but in our elephant population, the majority of the known-age individuals we focus on were still alive when our data recording ended. Including lifespan or ALR in our models would result in a biased sample of individuals who died young. Other methods use age-specific estimates of reproductive success and survival to determine the breeding success of individuals who do and do not survive to the next age to calculate the exact within-individual change between ages (Cam *et al*., 2002; Rebke *et al*., 2010), but these were unsuitable because we aimed to test for interactions between ageing and early life reproduction, which are incompatible with these methods. Therefore, we included censoring and age at last observation as fixed effects in our analyses. These were clearly important as they were statistically associated with annual breeding success, but despite their inclusion our results may not exactly describe individual-level ageing patterns. Second, we analyzed reproductive success of individuals of known birth date up until a maximum age of 53. Asian and African elephants (*L. africana*) are estimated to survive up to 80 years in the wild (Clubb *et al*., 2008), and individuals estimated to be aged over 60 have been recorded reproducing in the wild (Moss, 2001). Thus, we did not analyze reproduction and survival in the very oldest females, and nor did our estimates of ‘lifetime’ breeding success represent the entire lifespan in the majority of cases, although the number of calves produced by age 50 is extremely likely to be a reflection of the number of calves produced by age 70, as indicated by the positive association between early-life fecundity and breeding success up to age 50. These data are not alone in experiencing this shortcoming: other long-term data sets, such as the population of African elephants of Amboseli, Kenya, have not been established long enough to follow individuals from known birth date to a known death date of over 50 (Moss *et al*., 2011). Whereas elephants very rarely survive beyond 50 years in Western zoos (Clubb *et al*., 2008), individuals in the Myanmar and Amboseli populations have been estimated to be older than 50, but they are few in number (Moss *et al*., 2011) and their ages are estimated based on their size, tooth wear and tusk length at capture or first sighting (Moss, 2001) and thus are only approximate. Therefore, there is almost nothing known about reproductive rates at ages over 50 in wild elephants and despite these caveats, the results presented here are the most comprehensive analysis of age-specific reproductive success, trade-offs between early reproduction and later-life fitness, and selection on early fecundity in a long-lived mammal to date. Finally, the Myanmar timber elephants may experience different trade-offs between reproduction and survival compared to wild populations given that, on one hand, their workload increases stress and reduces foraging time but, on the other hand, scheduled ‘maternity leaves’ and human care could reduce the costs of reproduction. Thus, although mating opportunities are not managed, the process of gestation and weaning may be influenced by management practices, with work likely increasing and veterinary care decreasing stress of pregnancy and weaning compared to wild elephants. It currently therefore remains unknown how work and captivity affect calf survival to term and to weaning age, but both stillbirth (4%) and pre-weaning mortality (25.6%) in the population are considerably lower than those reported for zoo elephants (Mar *et al*., 2012) and more closely resemble those found in wild African elephants (Moss, 2001). Consequently, the Myanmar timber elephant dataset is uniquely detailed for a long-lived animal, and is still considered the best available information on Asian elephant life histories, or any species with similar longevity.

Our study has at least two implications. First, investigating senescence patterns in mammals which are comparable with other species studied so far in terms of life history but similar to humans in terms of lifespan, might elucidate some of the reasons for the evolution of unusual senescence pattern in human females (Williams, 1957; Lahdenperä *et al*., 2004, 2011, 2012). The evidence that high early reproductive effort in human females reduces later survival is contradictory (Helle *et al*., 2005), although the predicted negative relationships can be detected at the genetic level (Pettay *et al*., 2005). Furthermore, studies in humans investigating the effects of early reproductive investment on rates of reproductive senescence are generally lacking. The Myanmar timber elephants are among the few long-lived mammals for which extensive longitudinal data exists to provide a useful comparison in this respect. Second, Asian elephants are an iconic species with extreme conservation interest because of their impact on the ecosystem (Sukumar, 2003) and the declining numbers both in the wild and zoos (Sukumar, 1989; Wiese, 2000). Understanding age-specific changes in survival and reproduction therefore has important conservation and welfare implications. Our current understanding of elephant reproductive physiology and its changes with age is based on zoo populations, which experience high frequencies of obesity and an absence of males and natural hormonal cycling (Hermes *et al*., 2008). Increasing infertility in old zoo females has been attributed to failures to breed at early ages (before 30) and the subsequent reproductive track abnormalities and post-parturient problems arising from repetitive and continuous non-fertile oestrous cycles in early life (Hermes *et al*., 2008). Previous work on the Myanmar population analyzed survival and reproductive success in age groups at 5-year intervals to show that in early life there is a positive association between reproductive success and immediate survival, but that in later life, there is a negative association (Robinson *et al*., 2012). Our study builds on this by analyzing annual survival, reproductive success and calf survival to generate a clear picture of the trajectory of age-specific reproductive performance in this population. Accurately identifying the timing of improvement, peak and decline in reproductive performance provides important information for management of these endangered populations. In addition, we tested the prediction of evolutionary theory that Myanmar elephants that invested in early reproduction should pay a survival cost in later life. We found this to be the case, and suggest that this was possibly mediated by the fact that such females also reproduced frequently at older ages. Overall, we find that higher early-life fecundity was associated with lower later-life survival, but higher lifetime fitness. Thus, the earlier paper showed that reproduction in early life is associated with higher immediate survival, presumably because these individuals either have genes promoting high survival and fecundity in early life or experience high levels of resources and can invest in both (Robinson *et al*., 2012), while the current study shows that reproduction in early life is favoured by selection despite a survival cost in later life because of a positive association with breeding success in later life. Given that in the wild the Asian elephant is listed as endangered, understanding the factors which influence the ability of females to reproduce both in natural conditions and in captivity, and finding ways in which to mitigate any costs, will help to maintain wild elephant populations. Although we acknowledge that the costs of reproduction are likely to vary between populations depending, for example, on nutrition and workload, our results highlight the general importance of introducing well-planned birth spacing and provisioning for highly fecund young females to prevent adverse outcomes in later life.

In summary, our longitudinal study determined associations between early-life reproductive effort and later-life survival, reproductive success and their tradeoffs to show that ultimately, the strategy of high early reproductive investment leads to greater overall fitness despite its costs on later-life survival. Although such calculations are naturally sensitive to how early reproduction is defined and how large a proportion of the whole potential reproductive period such ‘early’ reproduction covers, the overall finding that the highest lifetime fitness was achieved by females with high early-life fecundity is of importance in understanding how the different reproductive strategies may evolve in natural populations where individuals reproduce across a long time-span and a range of environmental conditions. Our study also provides a much needed data point for comparisons of senescence in long-lived bird species that have slow life history with similarly long-lived species of mammals.

## Supporting Information

Table S1 A comparison of single-threshold models analyzing ageing-related variation in annual breeding success in females aged 5–50. **Table S2.** A comparison of two-threshold models analyzing ageing-related variation in annual breeding success in females aged 5–50. **Table S3.** A comparison of Cox proportional hazards models investigating survival following the age of 19 in individuals with different levels of early life fecundity (ELF).
