# Supplementary material for: Early reproductive investment, senescence and lifetime reproductive success in female Asian elephants
Source: J Evol Biol. 2014 Mar 3;27(4):772–83. doi: 10.1111/jeb.12350 (PMC4237172; doi:10.1111/jeb.12350)
Supplement: Table S1 — A comparison of single-threshold models analyzing ageing-related variation in annual breeding success in females aged 5–50. Table S2. A comparison of two-threshold models analyzing ageing-related variation in annual breeding success in females aged 5–50. Table S3. A comparison of Cox proportional hazards models investigating survival following the age of 19 in individuals with different levels of early life fecundity (ELF). [file jeb0027-0772-S3.docx]

**Supporting Tables**

**Table S1:** A comparison of single-threshold models analyzing ageing-related variation in annual breeding success in females aged 5-50. All models were generalized linear mixed-effects models (GLMMs) with binomial errors and logit link function. Models were compared using AIC values, where the best-supported model has the lowest AIC value. Models are shown in order of best fit, with the best-fitting model shown first; this model is shown as the best-fitting one-threshold model in Table 1 of the main text. ΔAIC values are shown relative to the best-supported model. Analysis was performed on 12,789 records from 416 female elephants.

| **Model** | **Threshold** | **AIC** | **ΔAIC** | **Model** | **Threshold** | **AIC** | **ΔAIC** |
| --- | --- | --- | --- | --- | --- | --- | --- |
| 17 | 17 | 7082.9 | 0.00 | 9 | 9 | 7304.3 | 221.38 |
| 16 | 16 | 7085.1 | 2.18 | 31 | 31 | 7315.2 | 232.36 |
| 18 | 18 | 7085.2 | 2.37 | 32 | 32 | 7331 | 248.14 |
| 19 | 19 | 7091.8 | 8.93 | 33 | 33 | 7346.4 | 263.53 |
| 15 | 15 | 7094.5 | 11.59 | 8 | 8 | 7355.2 | 272.28 |
| 20 | 20 | 7104.3 | 21.38 | 34 | 34 | 7367.5 | 284.64 |
| 14 | 14 | 7107 | 24.12 | 35 | 35 | 7386.9 | 304.02 |
| 21 | 21 | 7119.1 | 36.23 | 36 | 36 | 7392.7 | 309.86 |
| 22 | 22 | 7123.9 | 40.99 | 38 | 38 | 7415 | 332.08 |
| 13 | 13 | 7131.5 | 48.63 | 37 | 37 | 7415 | 332.12 |
| 12 | 12 | 7148 | 65.11 | 39 | 39 | 7425.4 | 342.52 |
| 23 | 23 | 7156 | 73.17 | 42 | 42 | 7440.8 | 357.94 |
| 24 | 24 | 7173.3 | 90.39 | 40 | 40 | 7441.5 | 358.62 |
| 25 | 25 | 7195.9 | 113.01 | 41 | 41 | 7442.2 | 359.31 |
| 26 | 26 | 7198.7 | 115.87 | 43 | 43 | 7454.6 | 371.73 |
| 11 | 11 | 7207.4 | 124.57 | 44 | 44 | 7460 | 377.14 |
| 27 | 27 | 7232.6 | 149.73 | 45 | 45 | 7474.3 | 391.39 |
| 10 | 10 | 7260.1 | 177.20 | 46 | 46 | 7477.1 | 394.24 |
| 28 | 28 | 7264.6 | 181.68 | 47 | 47 | 7479.6 | 396.77 |
| 29 | 29 | 7278.8 | 195.92 | 48 | 48 | 7485.4 | 402.53 |
| 30 | 30 | 7286.9 | 203.98 |  |  |  |  |

**Table S2 (below):** A comparison of two-threshold models analyzing ageing-related variation in annual breeding success in females aged 5-50. “T1” indicates where the first threshold ended and “T2” indicates where the second threshold ended. All models were generalized linear mixed-effects models (GLMMs) with binomial errors and logit link function. Models were compared using AIC values, where the best-supported model has the lowest AIC value. Models are shown in order of best fit, with the best-fitting model shown first; this model is shown as the best-fitting two-threshold model in Table 1 of the main text. ΔAIC values are shown relative to the best-supported model. Analysis was performed on 12,789 records from 416 female elephants.

| **Model** | **T1** | **T2** | **AIC** | **ΔAIC** | **Model** | **T1** | **T2** | **AIC** | **ΔAIC** |
| --- | --- | --- | --- | --- | --- | --- | --- | --- | --- |
| 1218 | 12 | 18 | 7075.92 | 0.00 | 1842 | 18 | 42 | 7085.65 | 9.73 |
| 1219 | 12 | 19 | 7076.37 | 0.45 | 1523 | 15 | 23 | 7085.8 | 9.88 |
| 1217 | 12 | 17 | 7076.96 | 1.04 | 1737 | 17 | 37 | 7085.86 | 9.94 |
| 1220 | 12 | 20 | 7078.59 | 2.67 | 1844 | 18 | 44 | 7086.01 | 10.09 |
| 1222 | 12 | 22 | 7079.12 | 3.20 | 1017 | 10 | 17 | 7086.02 | 10.11 |
| 1522 | 15 | 22 | 7080.63 | 4.71 | 1826 | 18 | 26 | 7086.03 | 10.12 |
| 1422 | 14 | 22 | 7081.07 | 5.16 | 1426 | 14 | 26 | 7086.08 | 10.17 |
| 1221 | 12 | 21 | 7081.34 | 5.42 | 1733 | 17 | 33 | 7086.1 | 10.19 |
| 1419 | 14 | 19 | 7081.54 | 5.62 | 1119 | 11 | 19 | 7086.11 | 10.19 |
| 1626 | 16 | 26 | 7081.64 | 5.73 | 1524 | 15 | 24 | 7086.2 | 10.28 |
| 1622 | 16 | 22 | 7081.69 | 5.78 | 1728 | 17 | 28 | 7086.25 | 10.34 |
| 1318 | 13 | 18 | 7081.8 | 5.89 | 1729 | 17 | 29 | 7086.26 | 10.35 |
| 1319 | 13 | 19 | 7082.27 | 6.36 | 1732 | 17 | 32 | 7086.27 | 10.36 |
| 1520 | 15 | 20 | 7082.33 | 6.41 | 1745 | 17 | 45 | 7086.31 | 10.39 |
| 1420 | 14 | 20 | 7082.45 | 6.54 | 1321 | 13 | 21 | 7086.34 | 10.42 |
| 1726 | 17 | 26 | 7082.6 | 6.69 | 1740 | 17 | 40 | 7086.37 | 10.45 |
| 1521 | 15 | 21 | 7082.9 | 6.98 | 1731 | 17 | 31 | 7086.37 | 10.46 |
| 1742 | 17 | 42 | 7082.97 | 7.06 | 1741 | 17 | 41 | 7086.44 | 10.52 |
| 1526 | 15 | 26 | 7083.19 | 7.28 | 1738 | 17 | 38 | 7086.49 | 10.57 |
| 1722 | 17 | 22 | 7083.4 | 7.48 | 1736 | 17 | 36 | 7086.53 | 10.61 |
| 1621 | 16 | 21 | 7083.41 | 7.49 | 1739 | 17 | 39 | 7086.56 | 10.64 |
| 1421 | 14 | 21 | 7083.47 | 7.55 | 1643 | 16 | 43 | 7086.57 | 10.65 |
| 1744 | 17 | 44 | 7083.53 | 7.61 | 1734 | 17 | 34 | 7086.65 | 10.74 |
| 1118 | 11 | 18 | 7083.9 | 7.99 | 1735 | 17 | 35 | 7086.7 | 10.78 |
| 1117 | 11 | 17 | 7083.93 | 8.02 | 1629 | 16 | 29 | 7086.88 | 10.97 |
| 1322 | 13 | 22 | 7083.97 | 8.06 | 1018 | 10 | 18 | 7086.95 | 11.04 |
| 1320 | 13 | 20 | 7084.19 | 8.27 | 1628 | 16 | 28 | 7087.02 | 11.11 |
| 1642 | 16 | 42 | 7084.49 | 8.57 | 1633 | 16 | 33 | 7087.16 | 11.24 |
| 1624 | 16 | 24 | 7084.74 | 8.83 | 1632 | 16 | 32 | 7087.26 | 11.34 |
| 1743 | 17 | 43 | 7084.74 | 8.83 | 1843 | 18 | 43 | 7087.26 | 11.35 |
| 1623 | 16 | 23 | 7084.75 | 8.83 | 1631 | 16 | 31 | 7087.28 | 11.37 |
| 1730 | 17 | 30 | 7084.89 | 8.97 | 1116 | 11 | 16 | 7087.39 | 11.47 |
| 1630 | 16 | 30 | 7085.14 | 9.23 | 1525 | 15 | 25 | 7087.56 | 11.65 |
| 1625 | 16 | 25 | 7085.23 | 9.31 | 1423 | 14 | 23 | 7087.66 | 11.74 |
| 1627 | 16 | 27 | 7085.38 | 9.47 | 1830 | 18 | 30 | 7087.94 | 12.02 |
| 1644 | 16 | 44 | 7085.44 | 9.52 | 1636 | 16 | 36 | 7088.01 | 12.10 |
| 1724 | 17 | 24 | 7085.45 | 9.53 | 1637 | 16 | 37 | 7088.02 | 12.11 |
| 1723 | 17 | 23 | 7085.48 | 9.56 | 1638 | 16 | 38 | 7088.06 | 12.14 |
| 1727 | 17 | 27 | 7085.5 | 9.58 | 1837 | 18 | 37 | 7088.06 | 12.15 |
| 1725 | 17 | 25 | 7085.58 | 9.66 | 1634 | 16 | 34 | 7088.07 | 12.16 |

| **Model** | **T1** | **T2** | **AIC** | **ΔAIC** | **Model** | **T1** | **T2** | **AIC** | **ΔAIC** |
| --- | --- | --- | --- | --- | --- | --- | --- | --- | --- |
| 1641 | 16 | 41 | 7088.18 | 12.26 | 1226 | 12 | 26 | 7093.21 | 17.29 |
| 1639 | 16 | 39 | 7088.24 | 12.32 | 1532 | 15 | 32 | 7093.33 | 17.41 |
| 1640 | 16 | 40 | 7088.47 | 12.55 | 1533 | 15 | 33 | 7093.5 | 17.58 |
| 1635 | 16 | 35 | 7088.47 | 12.56 | 1943 | 19 | 43 | 7093.88 | 17.97 |
| 1424 | 14 | 24 | 7088.57 | 12.66 | 1326 | 13 | 26 | 7094.02 | 18.10 |
| 1845 | 18 | 45 | 7088.58 | 12.66 | 1544 | 15 | 44 | 7094.2 | 18.28 |
| 1645 | 16 | 45 | 7088.6 | 12.69 | 1937 | 19 | 37 | 7094.45 | 18.54 |
| 1827 | 18 | 27 | 7088.62 | 12.70 | 1930 | 19 | 30 | 7094.72 | 18.81 |
| 1527 | 15 | 27 | 7088.63 | 12.72 | 1945 | 19 | 45 | 7095.05 | 19.14 |
| 1840 | 18 | 40 | 7088.68 | 12.77 | 1324 | 13 | 24 | 7095.13 | 19.22 |
| 1823 | 18 | 23 | 7088.69 | 12.78 | 1534 | 15 | 34 | 7095.13 | 19.22 |
| 1824 | 18 | 24 | 7088.73 | 12.81 | 1543 | 15 | 43 | 7095.14 | 19.22 |
| 1825 | 18 | 25 | 7088.74 | 12.82 | 1940 | 19 | 40 | 7095.17 | 19.25 |
| 1016 | 10 | 16 | 7088.75 | 12.84 | 1122 | 11 | 22 | 7095.39 | 19.48 |
| 1833 | 18 | 33 | 7088.83 | 12.91 | 1927 | 19 | 27 | 7095.41 | 19.49 |
| 1828 | 18 | 28 | 7088.92 | 13.01 | 1536 | 15 | 36 | 7095.44 | 19.52 |
| 1841 | 18 | 41 | 7088.98 | 13.06 | 1928 | 19 | 28 | 7095.47 | 19.56 |
| 1832 | 18 | 32 | 7089.01 | 13.10 | 1933 | 19 | 33 | 7095.49 | 19.57 |
| 1838 | 18 | 38 | 7089.07 | 13.15 | 1925 | 19 | 25 | 7095.53 | 19.62 |
| 1835 | 18 | 35 | 7089.08 | 13.16 | 1935 | 19 | 35 | 7095.55 | 19.64 |
| 1829 | 18 | 29 | 7089.08 | 13.17 | 1924 | 19 | 24 | 7095.57 | 19.65 |
| 1839 | 18 | 39 | 7089.1 | 13.18 | 1941 | 19 | 41 | 7095.6 | 19.69 |
| 1836 | 18 | 36 | 7089.1 | 13.19 | 1932 | 19 | 32 | 7095.67 | 19.75 |
| 1831 | 18 | 31 | 7089.11 | 13.20 | 1938 | 19 | 38 | 7095.69 | 19.78 |
| 1834 | 18 | 34 | 7089.21 | 13.29 | 1939 | 19 | 39 | 7095.7 | 19.79 |
| 1530 | 15 | 30 | 7089.68 | 13.76 | 1936 | 19 | 36 | 7095.71 | 19.79 |
| 1223 | 12 | 23 | 7089.97 | 14.05 | 1929 | 19 | 29 | 7095.75 | 19.84 |
| 1019 | 10 | 19 | 7090.58 | 14.67 | 1931 | 19 | 31 | 7095.76 | 19.84 |
| 1425 | 14 | 25 | 7090.82 | 14.90 | 1934 | 19 | 34 | 7095.78 | 19.86 |
| 1120 | 11 | 20 | 7091.06 | 15.15 | 1430 | 14 | 30 | 7095.79 | 19.88 |
| 1529 | 15 | 29 | 7091.78 | 15.87 | 1538 | 15 | 38 | 7095.84 | 19.93 |
| 1528 | 15 | 28 | 7091.91 | 15.99 | 1535 | 15 | 35 | 7096.21 | 20.30 |
| 1942 | 19 | 42 | 7092.35 | 16.43 | 1539 | 15 | 39 | 7096.29 | 20.38 |
| 1542 | 15 | 42 | 7092.5 | 16.59 | 1541 | 15 | 41 | 7096.49 | 20.57 |
| 1944 | 19 | 44 | 7092.61 | 16.69 | 1537 | 15 | 37 | 7096.62 | 20.70 |
| 1926 | 19 | 26 | 7092.83 | 16.91 | 1121 | 11 | 21 | 7096.62 | 20.71 |
| 1224 | 12 | 24 | 7092.89 | 16.98 | 1540 | 15 | 40 | 7097.28 | 21.36 |
| 1427 | 14 | 27 | 7093.05 | 17.13 | 1015 | 10 | 15 | 7097.63 | 21.72 |
| 1531 | 15 | 31 | 7093.06 | 17.14 | 1020 | 10 | 20 | 7097.71 | 21.79 |
| 1323 | 13 | 23 | 7093.14 | 17.23 | 1428 | 14 | 28 | 7097.88 | 21.97 |

| **Model** | **T1** | **T2** | **AIC** | **ΔAIC** | **Model** | **T1** | **T2** | **AIC** | **ΔAIC** |
| --- | --- | --- | --- | --- | --- | --- | --- | --- | --- |
| 1429 | 14 | 29 | 7098.01 | 22.09 | 2038 | 20 | 38 | 7108.19 | 32.27 |
| 1545 | 15 | 45 | 7098.02 | 22.10 | 2031 | 20 | 31 | 7108.2 | 32.28 |
| 1225 | 12 | 25 | 7098.03 | 22.11 | 1440 | 14 | 40 | 7108.67 | 32.76 |
| 1325 | 13 | 25 | 7098.94 | 23.03 | 1330 | 13 | 30 | 7109.28 | 33.36 |
| 1431 | 14 | 31 | 7100.43 | 24.52 | 1445 | 14 | 45 | 7110.35 | 34.44 |
| 1432 | 14 | 32 | 7101.13 | 25.22 | 1328 | 13 | 28 | 7110.58 | 34.67 |
| 1433 | 14 | 33 | 7101.72 | 25.80 | 1123 | 11 | 23 | 7110.98 | 35.07 |
| 1442 | 14 | 42 | 7103.33 | 27.41 | 1329 | 13 | 29 | 7111.34 | 35.43 |
| 1327 | 13 | 27 | 7103.39 | 27.47 | 1230 | 12 | 30 | 7113.69 | 37.78 |
| 1434 | 14 | 34 | 7104.16 | 28.24 | 1228 | 12 | 28 | 7113.87 | 37.96 |
| 1227 | 12 | 27 | 7104.6 | 28.68 | 1229 | 12 | 29 | 7115.4 | 39.48 |
| 2042 | 20 | 42 | 7104.97 | 29.05 | 1331 | 13 | 31 | 7115.82 | 39.91 |
| 1436 | 14 | 36 | 7105.09 | 29.18 | 1124 | 11 | 24 | 7116.41 | 40.50 |
| 2044 | 20 | 44 | 7105.1 | 29.19 | 1332 | 13 | 32 | 7117.34 | 41.43 |
| 1022 | 10 | 22 | 7105.58 | 29.66 | 1333 | 13 | 33 | 7118.7 | 42.78 |
| 1021 | 10 | 21 | 7105.62 | 29.71 | 3726 | 21 | 42 | 7119.93 | 44.02 |
| 2026 | 20 | 26 | 7105.82 | 29.91 | 3926 | 21 | 44 | 7119.97 | 44.05 |
| 1444 | 14 | 44 | 7105.86 | 29.94 | 1126 | 11 | 26 | 7120.51 | 44.59 |
| 1435 | 14 | 35 | 7106.01 | 30.10 | 2126 | 21 | 26 | 7120.96 | 45.04 |
| 1438 | 14 | 38 | 7106.08 | 30.17 | 3226 | 21 | 37 | 7121.09 | 45.17 |
| 2043 | 20 | 43 | 7106.39 | 30.48 | 3826 | 21 | 43 | 7121.27 | 45.36 |
| 1443 | 14 | 43 | 7106.55 | 30.64 | 2326 | 21 | 28 | 7121.69 | 45.78 |
| 2037 | 20 | 37 | 7106.59 | 30.68 | 1231 | 12 | 31 | 7122.02 | 46.11 |
| 1439 | 14 | 39 | 7106.88 | 30.97 | 4026 | 21 | 45 | 7122.12 | 46.20 |
| 2045 | 20 | 45 | 7107.38 | 31.47 | 3526 | 21 | 40 | 7122.16 | 46.25 |
| 2030 | 20 | 30 | 7107.44 | 31.53 | 3026 | 21 | 35 | 7122.35 | 46.43 |
| 1437 | 14 | 37 | 7107.47 | 31.56 | 2526 | 21 | 30 | 7122.37 | 46.45 |
| 2040 | 20 | 40 | 7107.48 | 31.56 | 1334 | 13 | 34 | 7122.41 | 46.49 |
| 1441 | 14 | 41 | 7107.51 | 31.60 | 2226 | 21 | 27 | 7122.69 | 46.77 |
| 2028 | 20 | 28 | 7107.6 | 31.68 | 2426 | 21 | 29 | 7122.72 | 46.80 |
| 2035 | 20 | 35 | 7107.79 | 31.88 | 2826 | 21 | 33 | 7122.76 | 46.84 |
| 2033 | 20 | 33 | 7107.99 | 32.07 | 2926 | 21 | 34 | 7122.81 | 46.90 |
| 2027 | 20 | 27 | 7108.04 | 32.13 | 2626 | 21 | 31 | 7122.85 | 46.94 |
| 2025 | 20 | 25 | 7108.09 | 32.18 | 2726 | 21 | 32 | 7122.87 | 46.95 |
| 2041 | 20 | 41 | 7108.11 | 32.20 | 3126 | 21 | 36 | 7122.93 | 47.01 |
| 2032 | 20 | 32 | 7108.14 | 32.23 | 3626 | 21 | 41 | 7122.99 | 47.07 |
| 2034 | 20 | 34 | 7108.15 | 32.23 | 3426 | 21 | 39 | 7123.02 | 47.10 |
| 2036 | 20 | 36 | 7108.16 | 32.24 | 3326 | 21 | 38 | 7123.03 | 47.11 |
| 2029 | 20 | 29 | 7108.17 | 32.26 | 2242 | 22 | 42 | 7124.21 | 48.30 |
| 2039 | 20 | 39 | 7108.19 | 32.27 | 1232 | 12 | 32 | 7124.45 | 48.53 |

| **Model** | **T1** | **T2** | **AIC** | **ΔAIC** | **Model** | **T1** | **T2** | **AIC** | **ΔAIC** |
| --- | --- | --- | --- | --- | --- | --- | --- | --- | --- |
| 1336 | 13 | 36 | 7124.55 | 48.64 | 1026 | 10 | 26 | 7140.85 | 64.93 |
| 2244 | 22 | 44 | 7124.63 | 48.72 | 1237 | 12 | 37 | 7140.93 | 65.01 |
| 1125 | 11 | 25 | 7124.64 | 48.72 | 1241 | 12 | 41 | 7143.07 | 67.15 |
| 1023 | 10 | 23 | 7125.07 | 49.15 | 1243 | 12 | 43 | 7143.67 | 67.75 |
| 1342 | 13 | 42 | 7125.16 | 49.24 | 1244 | 12 | 44 | 7143.81 | 67.90 |
| 1335 | 13 | 35 | 7125.45 | 49.54 | 1025 | 10 | 25 | 7143.97 | 68.06 |
| 2243 | 22 | 43 | 7125.88 | 49.97 | 1240 | 12 | 40 | 7144.75 | 68.84 |
| 2230 | 22 | 30 | 7126.31 | 50.40 | 1128 | 11 | 28 | 7149.11 | 73.20 |
| 2237 | 22 | 37 | 7126.52 | 50.61 | 1245 | 12 | 45 | 7150.12 | 74.20 |
| 1338 | 13 | 38 | 7126.64 | 50.73 | 1130 | 11 | 30 | 7151.59 | 75.67 |
| 1233 | 12 | 33 | 7126.66 | 50.75 | 1129 | 11 | 29 | 7152.3 | 76.38 |
| 2245 | 22 | 45 | 7127.14 | 51.23 | 2342 | 23 | 42 | 7156.81 | 80.89 |
| 2227 | 22 | 27 | 7127.16 | 51.24 | 2344 | 23 | 44 | 7156.88 | 80.96 |
| 2240 | 22 | 40 | 7127.26 | 51.35 | 2337 | 23 | 37 | 7157.96 | 82.05 |
| 2228 | 22 | 28 | 7127.4 | 51.48 | 2343 | 23 | 43 | 7158.19 | 82.27 |
| 2233 | 22 | 33 | 7127.46 | 51.54 | 2328 | 23 | 28 | 7158.26 | 82.35 |
| 2241 | 22 | 41 | 7127.61 | 51.69 | 2345 | 23 | 45 | 7159.03 | 83.11 |
| 2235 | 22 | 35 | 7127.64 | 51.72 | 2340 | 23 | 40 | 7159.1 | 83.18 |
| 2232 | 22 | 32 | 7127.65 | 51.73 | 2330 | 23 | 30 | 7159.18 | 83.26 |
| 2229 | 22 | 29 | 7127.69 | 51.78 | 2335 | 23 | 35 | 7159.24 | 83.32 |
| 2238 | 22 | 38 | 7127.7 | 51.79 | 2329 | 23 | 29 | 7159.66 | 83.75 |
| 2239 | 22 | 39 | 7127.73 | 51.81 | 2333 | 23 | 33 | 7159.67 | 83.76 |
| 2236 | 22 | 36 | 7127.74 | 51.83 | 2334 | 23 | 34 | 7159.73 | 83.81 |
| 2231 | 22 | 31 | 7127.76 | 51.84 | 2331 | 23 | 31 | 7159.78 | 83.86 |
| 2234 | 22 | 34 | 7127.84 | 51.93 | 2332 | 23 | 32 | 7159.79 | 83.88 |
| 1339 | 13 | 39 | 7128.04 | 52.13 | 2336 | 23 | 36 | 7159.86 | 83.95 |
| 1337 | 13 | 37 | 7128.63 | 52.72 | 2341 | 23 | 41 | 7159.92 | 84.00 |
| 1344 | 13 | 44 | 7128.95 | 53.03 | 1027 | 10 | 27 | 7159.93 | 84.02 |
| 1343 | 13 | 43 | 7129.24 | 53.33 | 2339 | 23 | 39 | 7159.95 | 84.04 |
| 1341 | 13 | 41 | 7129.49 | 53.57 | 2338 | 23 | 38 | 7159.96 | 84.05 |
| 1340 | 13 | 40 | 7130.99 | 55.07 | 1131 | 11 | 31 | 7163.08 | 87.17 |
| 1234 | 12 | 34 | 7131.64 | 55.73 | 1132 | 11 | 32 | 7167.25 | 91.33 |
| 1024 | 10 | 24 | 7132.87 | 56.95 | 1133 | 11 | 33 | 7171.12 | 95.20 |
| 1345 | 13 | 45 | 7134.36 | 58.44 | 2442 | 24 | 42 | 7173.95 | 98.03 |
| 1236 | 12 | 36 | 7135.2 | 59.29 | 2444 | 24 | 44 | 7174.08 | 98.17 |
| 1235 | 12 | 35 | 7135.9 | 59.99 | 2437 | 24 | 37 | 7175.21 | 99.29 |
| 1127 | 11 | 27 | 7135.95 | 60.03 | 2443 | 24 | 43 | 7175.4 | 99.48 |
| 1238 | 12 | 38 | 7138.58 | 62.67 | 2445 | 24 | 45 | 7176.27 | 100.35 |
| 1242 | 12 | 42 | 7138.73 | 62.82 | 2440 | 24 | 40 | 7176.35 | 100.43 |
| 1239 | 12 | 39 | 7140.65 | 64.73 | 2430 | 24 | 30 | 7176.37 | 100.45 |

| **Model** | **T1** | **T2** | **AIC** | **ΔAIC** | **Model** | **T1** | **T2** | **AIC** | **ΔAIC** |
| --- | --- | --- | --- | --- | --- | --- | --- | --- | --- |
| 2435 | 24 | 35 | 7176.48 | 100.57 | 2539 | 25 | 39 | 7199.77 | 123.85 |
| 1028 | 10 | 28 | 7176.68 | 100.76 | 2538 | 25 | 38 | 7199.78 | 123.87 |
| 2429 | 24 | 29 | 7176.87 | 100.96 | 2643 | 26 | 43 | 7200.59 | 124.67 |
| 2433 | 24 | 33 | 7176.92 | 101.01 | 1144 | 11 | 44 | 7200.64 | 124.72 |
| 2434 | 24 | 34 | 7176.98 | 101.07 | 2637 | 26 | 37 | 7201.47 | 125.55 |
| 2431 | 24 | 31 | 7177.03 | 101.11 | 2633 | 26 | 33 | 7201.83 | 125.91 |
| 2432 | 24 | 32 | 7177.05 | 101.13 | 2632 | 26 | 32 | 7201.94 | 126.03 |
| 2436 | 24 | 36 | 7177.11 | 101.19 | 2631 | 26 | 31 | 7202.01 | 126.09 |
| 2441 | 24 | 41 | 7177.13 | 101.21 | 2645 | 26 | 45 | 7202.14 | 126.23 |
| 2439 | 24 | 39 | 7177.18 | 101.26 | 2640 | 26 | 40 | 7202.2 | 126.28 |
| 2438 | 24 | 38 | 7177.19 | 101.27 | 2641 | 26 | 41 | 7202.23 | 126.32 |
| 1134 | 11 | 34 | 7178.34 | 102.43 | 2638 | 26 | 38 | 7202.24 | 126.33 |
| 1029 | 10 | 29 | 7181.63 | 105.71 | 2636 | 26 | 36 | 7202.35 | 126.43 |
| 1030 | 10 | 30 | 7182.16 | 106.24 | 2639 | 26 | 39 | 7202.36 | 126.44 |
| 1136 | 11 | 36 | 7184.57 | 108.65 | 2635 | 26 | 35 | 7202.48 | 126.56 |
| 1135 | 11 | 35 | 7184.69 | 108.78 | 2634 | 26 | 34 | 7202.56 | 126.64 |
| 1138 | 11 | 38 | 7190.3 | 114.38 | 1032 | 10 | 32 | 7202.64 | 126.72 |
| 1137 | 11 | 37 | 7192.87 | 116.95 | 1145 | 11 | 45 | 7208.25 | 132.34 |
| 1142 | 11 | 42 | 7193.5 | 117.58 | 1033 | 10 | 33 | 7208.29 | 132.38 |
| 1139 | 11 | 39 | 7193.5 | 117.59 | 1034 | 10 | 34 | 7217.79 | 141.87 |
| 2542 | 25 | 42 | 7196.65 | 120.73 | 1035 | 10 | 35 | 7226.24 | 150.33 |
| 1031 | 10 | 31 | 7196.65 | 120.73 | 1036 | 10 | 36 | 7226.86 | 150.94 |
| 2544 | 25 | 44 | 7196.72 | 120.80 | 2742 | 27 | 42 | 7232.76 | 156.84 |
| 2537 | 25 | 37 | 7197.45 | 121.53 | 2744 | 27 | 44 | 7233.35 | 157.44 |
| 1141 | 11 | 41 | 7197.69 | 121.77 | 2743 | 27 | 43 | 7234.61 | 158.69 |
| 2543 | 25 | 43 | 7198.03 | 122.11 | 2737 | 27 | 37 | 7235.03 | 159.11 |
| 2642 | 26 | 42 | 7198.56 | 122.65 | 1038 | 10 | 38 | 7235.07 | 159.15 |
| 2535 | 25 | 35 | 7198.72 | 122.81 | 2745 | 27 | 45 | 7235.81 | 159.90 |
| 2545 | 25 | 45 | 7198.79 | 122.87 | 2740 | 27 | 40 | 7235.98 | 160.06 |
| 2540 | 25 | 40 | 7198.81 | 122.90 | 2733 | 27 | 33 | 7236.15 | 160.24 |
| 2530 | 25 | 30 | 7198.95 | 123.04 | 2735 | 27 | 35 | 7236.25 | 160.33 |
| 2531 | 25 | 31 | 7199.18 | 123.26 | 2741 | 27 | 41 | 7236.3 | 160.39 |
| 2534 | 25 | 34 | 7199.31 | 123.40 | 2732 | 27 | 32 | 7236.32 | 160.41 |
| 2533 | 25 | 33 | 7199.37 | 123.46 | 2738 | 27 | 38 | 7236.38 | 160.46 |
| 2644 | 26 | 44 | 7199.39 | 123.48 | 2739 | 27 | 39 | 7236.43 | 160.51 |
| 2532 | 25 | 32 | 7199.39 | 123.48 | 2736 | 27 | 36 | 7236.47 | 160.55 |
| 1140 | 11 | 40 | 7199.41 | 123.50 | 2734 | 27 | 34 | 7236.59 | 160.68 |
| 2536 | 25 | 36 | 7199.62 | 123.70 | 1037 | 10 | 37 | 7237.57 | 161.66 |
| 2541 | 25 | 41 | 7199.76 | 123.84 | 1039 | 10 | 39 | 7239.4 | 163.48 |
| 1143 | 11 | 43 | 7199.77 | 123.85 | 1042 | 10 | 42 | 7241.54 | 165.62 |

| **Model** | **T1** | **T2** | **AIC** | **ΔAIC** | **Model** | **T1** | **T2** | **AIC** | **ΔAIC** |
| --- | --- | --- | --- | --- | --- | --- | --- | --- | --- |
| 1041 | 10 | 41 | 7245.45 | 169.54 | 3036 | 30 | 36 | 7290.44 | 214.52 |
| 1040 | 10 | 40 | 7247.03 | 171.11 | 3142 | 31 | 42 | 7315.08 | 239.16 |
| 1043 | 10 | 43 | 7249.09 | 173.17 | 3144 | 31 | 44 | 7315.94 | 240.02 |
| 1044 | 10 | 44 | 7250.67 | 174.76 | 3137 | 31 | 37 | 7316.9 | 240.99 |
| 1045 | 10 | 45 | 7259.47 | 183.55 | 3143 | 31 | 43 | 7317.18 | 241.26 |
| 2842 | 28 | 42 | 7265.18 | 189.26 | 3145 | 31 | 45 | 7318.27 | 242.36 |
| 2844 | 28 | 44 | 7265.35 | 189.43 | 3140 | 31 | 40 | 7318.53 | 242.61 |
| 2837 | 28 | 37 | 7265.64 | 189.72 | 3141 | 31 | 41 | 7318.88 | 242.96 |
| 2843 | 28 | 43 | 7266.66 | 190.74 | 3138 | 31 | 38 | 7318.97 | 243.05 |
| 2835 | 28 | 35 | 7266.84 | 190.92 | 3139 | 31 | 39 | 7319.03 | 243.12 |
| 2845 | 28 | 45 | 7267.32 | 191.40 | 3136 | 31 | 36 | 7319.14 | 243.22 |
| 2840 | 28 | 40 | 7267.4 | 191.48 | 3242 | 32 | 42 | 7330.46 | 254.54 |
| 2834 | 28 | 34 | 7267.53 | 191.61 | 3244 | 32 | 44 | 7331.67 | 255.76 |
| 2833 | 28 | 33 | 7267.68 | 191.76 | 3237 | 32 | 37 | 7332.81 | 256.89 |
| 2836 | 28 | 36 | 7268.16 | 192.25 | 3243 | 32 | 43 | 7332.86 | 256.95 |
| 2839 | 28 | 39 | 7268.4 | 192.48 | 3245 | 32 | 45 | 7334.16 | 258.24 |
| 2841 | 28 | 41 | 7268.4 | 192.48 | 3240 | 32 | 40 | 7334.38 | 258.47 |
| 2838 | 28 | 38 | 7268.41 | 192.50 | 3238 | 32 | 38 | 7334.42 | 258.51 |
| 2942 | 29 | 42 | 7279.22 | 203.30 | 3241 | 32 | 41 | 7334.47 | 258.55 |
| 2944 | 29 | 44 | 7279.59 | 203.68 | 3239 | 32 | 39 | 7334.63 | 258.71 |
| 2937 | 29 | 37 | 7280.06 | 204.14 | 3342 | 33 | 42 | 7345.24 | 269.33 |
| 2943 | 29 | 43 | 7280.88 | 204.96 | 3344 | 33 | 44 | 7346.98 | 271.06 |
| 2935 | 29 | 35 | 7281.2 | 205.28 | 3343 | 33 | 43 | 7348.09 | 272.18 |
| 2945 | 29 | 45 | 7281.69 | 205.77 | 3338 | 33 | 38 | 7348.88 | 272.97 |
| 2940 | 29 | 40 | 7281.82 | 205.90 | 3341 | 33 | 41 | 7349.47 | 273.55 |
| 2934 | 29 | 34 | 7281.91 | 205.99 | 3339 | 33 | 39 | 7349.5 | 273.59 |
| 2936 | 29 | 36 | 7282.57 | 206.66 | 3345 | 33 | 45 | 7349.68 | 273.77 |
| 2941 | 29 | 41 | 7282.62 | 206.71 | 3340 | 33 | 40 | 7349.77 | 273.86 |
| 2939 | 29 | 39 | 7282.69 | 206.78 | 3442 | 34 | 42 | 7366.58 | 290.66 |
| 2938 | 29 | 38 | 7282.7 | 206.79 | 3444 | 34 | 44 | 7368.15 | 292.23 |
| 3042 | 30 | 42 | 7286.29 | 210.37 | 3443 | 34 | 43 | 7369.33 | 293.42 |
| 3044 | 30 | 44 | 7287.49 | 211.57 | 3445 | 34 | 45 | 7370.55 | 294.63 |
| 3043 | 30 | 43 | 7288.65 | 212.73 | 3441 | 34 | 41 | 7370.79 | 294.88 |
| 3037 | 30 | 37 | 7289.15 | 213.24 | 3439 | 34 | 39 | 7370.83 | 294.92 |
| 3038 | 30 | 38 | 7290.16 | 214.25 | 3440 | 34 | 40 | 7370.88 | 294.96 |
| 3045 | 30 | 45 | 7290.19 | 214.28 | 3542 | 35 | 42 | 7386.52 | 310.61 |
| 3041 | 30 | 41 | 7290.21 | 214.30 | 3544 | 35 | 44 | 7387.5 | 311.59 |
| 3040 | 30 | 40 | 7290.27 | 214.36 | 3543 | 35 | 43 | 7388.8 | 312.88 |
| 3035 | 30 | 35 | 7290.31 | 214.40 | 3545 | 35 | 45 | 7389.29 | 313.37 |
| 3039 | 30 | 39 | 7290.35 | 214.43 | 3540 | 35 | 40 | 7389.74 | 313.82 |

| **Model** | **T1** | **T2** | **AIC** | **ΔAIC** | **Model** | **T1** | **T2** | **AIC** | **ΔAIC** |
| --- | --- | --- | --- | --- | --- | --- | --- | --- | --- |
| 3541 | 35 | 41 | 7390.54 | 314.63 | 3745 | 37 | 45 | 7415.57 | 339.66 |
| 3632 | 36 | 42 | 7391.09 | 315.17 | 3844 | 38 | 44 | 7415.81 | 339.90 |
| 3634 | 36 | 44 | 7393.42 | 317.51 | 3743 | 37 | 43 | 7416.19 | 340.28 |
| 3633 | 36 | 43 | 7394.55 | 318.64 | 3843 | 38 | 43 | 7417.05 | 341.14 |
| 3631 | 36 | 41 | 7395.48 | 319.57 | 3845 | 38 | 45 | 7417.33 | 341.42 |
| 3635 | 36 | 45 | 7395.74 | 319.83 | 3944 | 39 | 44 | 7426.26 | 350.35 |
| 3742 | 37 | 42 | 7414.61 | 338.69 | 3945 | 39 | 45 | 7427.53 | 351.62 |
| 3744 | 37 | 44 | 7414.86 | 338.94 | 4045 | 40 | 45 | 7439.01 | 363.10 |

**Table S3:** A comparison of Cox proportional hazards models investigating survival following the age of 19 in individuals with different levels of early life fecundity (ELF). Model 0 fits the same hazard function to individuals who produced 0, 1 or 2 calves before age 19, while the remaining models fit different functions for individuals of different levels of early-life reproductive investment.

| **Model** | **Structure** | **LogLik** | **χ²** | **vs.** | **d.f.** | **p** |
| --- | --- | --- | --- | --- | --- | --- |
| 0 | ELF 0, 1, 2 | -443.98 |  |  |  |  |
| 1 | ELF 0 versus 1 versus 2 | -439.55 | 8.86 | 0 | 2 | 0.012 |
| 2 | ELF 0 versus 1 + 2 | -439.60 | 8.76 | 1 | 1 | 0.003 |
| 3 | ELF 0 + 1 versus 2 | -443.30 | 1.36 | 0 | 1 | 0.244 |
